# Generation of 2,000 breast cancer metabolic landscapes reveals a poor prognosis group with active serotonin production

**DOI:** 10.1038/srep19771

**Published:** 2016-01-27

**Authors:** Vytautas Leoncikas, Huihai Wu, Lara T. Ward, Andrzej M. Kierzek, Nick J. Plant

**Affiliations:** 1 School of Bioscience and Medicine, Faculty of Health and Medical Sciences, University of Surrey, Guildford, Surrey, GU2 7XH, United Kingdom; 2 Oncology DMPK, AstraZeneca, Alderley Park, Cheshire, SK10 4TG, United Kingdom

## Abstract

A major roadblock in the effective treatment of cancers is their heterogeneity, whereby multiple molecular landscapes are classified as a single disease. To explore the contribution of cellular metabolism to cancer heterogeneity, we analyse the Metabric dataset, a landmark genomic and transcriptomic study of 2,000 individual breast tumours, in the context of the human genome-scale metabolic network. We create personalized metabolic landscapes for each tumour by exploring sets of active reactions that satisfy constraints derived from human biochemistry and maximize congruency with the Metabric transcriptome data. Classification of the personalized landscapes derived from 997 tumour samples within the Metabric discovery dataset reveals a novel poor prognosis cluster, reproducible in the 995-sample validation dataset. We experimentally follow mechanistic hypotheses resulting from the computational study and establish that active serotonin production is a major metabolic feature of the poor prognosis group. These data support the reconsideration of concomitant serotonin-specific uptake inhibitors treatment during breast cancer chemotherapy.

A major challenge in breast cancer diagnostics and therapy is the heterogeneous nature of tumours, whereby multiple molecular landscapes are classified as a single disease. These diverse tumour phenotypes are unlikely to respond in a similar manner to therapeutic intervention, often leading to sub-optimal patient response. Effective patient stratification and treatment is currently hindered by the lack of clear consensus on breast tumour classification^1^. The development of molecular classification approaches provided a potentially superior methodology to traditional histological taxonomy. However, as with histological taxonomy, there is no clear definition of the optimal number of molecular groups, or robust methods to perform molecular classification in a clinical setting^2^,^3^. These issues are highlighted by the fact that in the past decade three single-sample predictor (SSP) methods were published yet little agreement exists between them^1^. As a result, neither histological nor molecular classifications accurately predict clinical outcome alone^3^; rather, they are often used in a complementary fashion during tumour stratification. On this basis, breast tumours are commonly classified into three clinically significant groups: hormone sensitive, Her2 positive and triple negative breast cancers (TNBC)^4^. This provides adequate therapeutic classification for hormone sensitive and Her2-positive tumours, where good therapeutic options exist. However, TNBC tumours are characterized by a lack of molecular targets and tendency to develop drug resistance, and correspondingly represent the leading cause of death in breast cancer^5^. It is thus imperative that novel approaches are used to further classify TNBCs, aiding the development of improved stratification and therapeutic options that would exploit tumour vulnerabilities, mitigate drug resistance and lead to improved patient response.

In a landmark study towards the molecular stratification of breast cancer, Metabric (Molecular Taxonomy of Breast Cancer International Consortium) collected over 2,000 clinically annotated, fresh-frozen breast cancer specimens from biobanks in the UK and Canada^2^. The individual tumours were subsequently subjected to transcriptomic and genomic profiling, leading to an unprecedented legacy dataset, which we will refer to as the Metabric dataset. This dataset is composed of two independent datasets: a discovery set (997 tumours) used for initial analysis, and a validation set (995 tumours) used to cross-validate findings.

In the original Metabric publication^2^ the molecular landscapes of breast cancer subgroups were characterized by standard statistical approaches based on over-representation of functional gene descriptors. However, emerging evidence suggests that such analysis may not fully exploit the value of clinical high throughput datasets. Instead, additional constraints imposed through the application of molecular network analysis are likely to increase the robustness, and biological relevance, of predictions^6^,^7^,^8^,^9^. Constraint based modeling (CBM) of Genome Scale Metabolic Networks (GSMNs) provides a well-established approach to examine the relationship between genotype and metabolic phenotype^9^,^10^. Rather than identifying statistical associations between gene-centered data and phenotype, CBM predicts the metabolic phenotype through simulation of the GSMN, a mathematical model of the network of coupled biochemical reactions derived from the repertoire of enzymes encoded in the genome (genotype). The GSMN is used to formulate constraints reflecting reaction stoichiometry and thermodynamics, and the space of metabolic flux distributions that satisfy these stoichiometric and thermodynamic constraints is then exploited^10^. Recently, the Recon 2 reconstruction of a general human GSMN has been published^11^. This represents the most comprehensive human GSMN to date, and has been thoroughly validated through its ability to robustly reproduce both inborn errors of metabolism and the exametabolome of the NCI 60 cancer cell line resource^11^. This general reconstruction of the human biochemical reaction network can be further constrained by clinical transcriptomics datasets to derive sample-specific GSMNs, reflecting not only the repertoire of enzymes encoded by the human genome, but also the expression of these genes in particular clinical sample^9^. As such, this approach has the potential to lead to significantly enhanced biological understanding from legacy datasets such as the Metabric dataset. Indeed, a CBM has been already successfully applied to study metabolic reprogramming in cancer and provide a mechanistic interpretation of cancer cell line-specific transcriptome datasets. Yizhak *et al.* have previously analyzed breast cancer transcriptome data in the context of Recon 1 GSMN^12^,^13^; however this study generated metabolic landscapes based on transformed cell lines, rather than clinical samples. The authors then used these models to identify metabolic genes of interest, which they then examined within the Metabric dataset. Yizhak *et al.* did not generate metabolic landscapes for all individual Metabric samples, and were hence unable to relate personalized features of tumour metabolism to clinical information available for each of the tumours. This was due to the limitation of their computational approach requiring information about tumour cell growth rate, which is readily available for cell lines, but not clinical samples. The advances in this field have been comprehensively reviewed in the recent article of Yizhak and colleagues^14^.

Here, we for the first time analyze the entire Metabric dataset in the context of the human GSMN Recon 2 and generate sample-specific GSMNs for all 2,000 transcriptomic datasets. Classification of these personalized metabolic landscapes derived from the Metabric discovery set reveals a novel poor prognosis subgroup, which is reproduced in the independent validation dataset. Analysis of the metabolic landscapes belonging to this subgroup provides several mechanistic hypotheses that we experimentally investigate. We demonstrate that active serotonin production is a major metabolic feature of the poor prognosis subgroup, and provide a potential molecular mechanism by which serotonin may promote cell viability in breast cancer cell lines. The association between active serotonin production and poor patient prognosis warrants further clinical examination, including a re-examining of the use of selective serotonin re-uptake inhibitors (SSRIs) in this group of patients.

## Results

### Classification of personalized metabolic landscapes reveals a novel poor patient prognosis cluster

**Figure 1:**
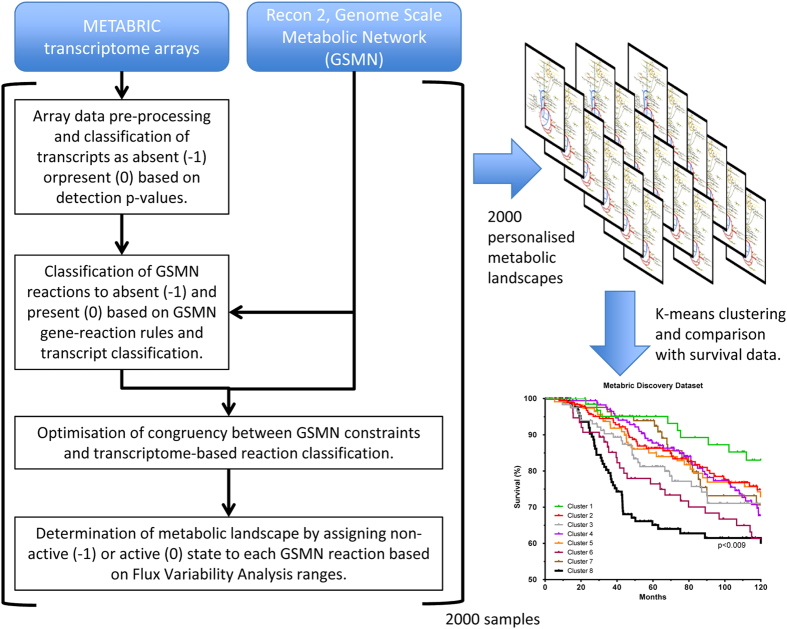
Calculation and classification of personalized metabolic landscapes. Transcriptome data from METABRICK dataset were pre-processed following original protocol. The Illumina detection p-values were used to classify each transcript as absent or present, the states encoded by −1 and 0 respectively. Subsequently, transcript classification and gene-reaction association rules of Recon 2 Genome Scale Metabolic Network (GSMN) were used to calculate reaction states of −1 or 0. In next step, Mixed Integer Linear Programming (MILP) optimization of congruency between transcriptome-based reaction states and GSMN constraints has been performed. Finally, Flux Variability Analysis of the model constrained by the MILP solution was used to classify reactions as non-active (−1) or active (0); we refer to a vector of reaction activities as metabolic landscape. Metabolic landscapes were subjected to k-means clustering and related to survival data.

To identify cellular metabolic activities associated with poor patient prognosis we have analyzed the transcriptome of the 997-sample discovery dataset in the context of the human GSMN Recon 2. The Recon 2 model represents a complete set of reactions encoded by the human genome. In a healthy tissue or tumour only a subset of all metabolic enzyme genes is expressed and only a subset of metabolic reactions active. Thus, for each transcriptome sample in the discovery set, we have created a sample-specific version of Recon 2, where transcriptome data were used to determine which metabolic reactions are not active in a particular tumour. Figure 1 provides an outline of the analysis pipeline, with further details of the computational protocol given in Methods and Supplementary Information. Briefly, we searched for GSMNs that satisfy the stoichiometric and thermodynamic constraints of Recon 2 while maximizing congruency of non-active reaction assignments with the transcriptomics data. We first used Illumina detection calls to identify genes encoding metabolic enzymes that are not expressed (absent) in a particular sample. Subsequently, alternate sample-specific GSMNs were explored to select the model with the maximal number of reactions associated with absent transcripts being declared non-active. Finally, all non-active reactions in the Recon 2 model were assigned an activity of −1, with other reactions assigned an activity of 0. We will refer to the set of metabolic reaction activities obtained for each particular tumour as its personalized metabolic landscape.

**Figure 2:**
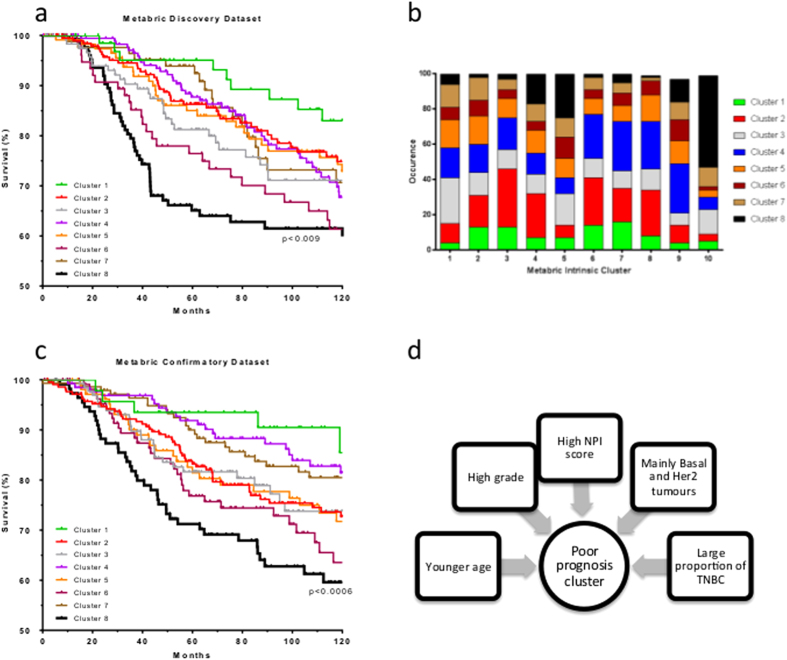
Identification of the metabolic landscape of breast tumours associated with poor patient prognosis. (**a**) Personalized GSMNs were derived from the 997 patient transcriptome profiles within the Metabric discovery dataset. A Kaplan-Mayer survival probability plot identifies a cluster associated with poor patient prognosis. (**b**) Distribution of personalized GSMNs from this analysis (clusters 1–8) against intrinsic clusters identified within the original Metabric publications. (**c**) Personalized GSMNs derived for the 995 patient transcriptome profiles within the Metabric validation dataset reproduces a cluster associated with poor patient prognosis, with a 99% congruency in differentially activated reactions. (**c**) Personalized GSMNs within the poor patient prognosis cluster are derived from patients with clinical characteristics that are previously associated with poor patient prognosis.

We next applied K-means clustering to classify these 997 personalized metabolic landscapes derived from the Metabric discovery dataset. This classification resulted in clusters of patients grouped by the activities of 7440 metabolic reactions; of these, the activity state of 3016 reactions varied between the clusters, adding considerable biological constraint compared to analysis of the raw transcriptome data alone. Since clinical follow-up data were available for each of the patients, we calculated a survival curve for each cluster. Alternative classifications with a range of clusters from 5 to 10 were examined, with eight clusters determined optimal for patient survival stratification (see Methods). As demonstrated in Fig. 2a, cluster 8 is comprised of 130 metabolic landscapes and has a statistically significant association with poor patient survival, (p < 0.009). We compared the distribution of the clusters based upon the individual metabolic landscapes presented in our work, with those based solely on transcriptomic data in the original publication^2^. Personalized metabolic landscapes within the poor prognosis cluster identified in our analysis (cluster 8), are derived from transcriptome data primarily found in the Metabric intrinsic clusters 5 and 10, the former of which is identified in the original publication as a poor-prognosis cluster.

Within the Metabric study, two fully independent sets of clinical samples were used to generate the discovery and validation datasets. Each data set contains data from approximately 1000 patients, and were used for independent validation of study findings^2^. Personalized metabolic landscapes were computed for the 995 patients in the validation dataset, and subject to K-means clustering as previously. The optimal classification to 8 clusters was reproduced, with 3471/7440 reactions having variable activity states across these clusters. In addition, the existence of a statistically significant cluster associated with poor patient prognosis was reproduced in this independent dataset (p < 0.0006; Fig. 2b). In the validation dataset, this poor prognosis cluster 8 was comprised 108/995 patients (11% of dataset), consistent with the 130/997 (13% of dataset) identified within the discovery dataset (p = 0.95). Critically, within the personalized metabolic landscapes belonging to the poor patient prognosis clusters derived from the discovery and validation datasets, 2199/2275 (97%) of the reactions that defined these clusters had the same activity states (Table S1), demonstrating the reproducibility of this poor prognosis cluster across the two independent datasets. Patients within the poor prognosis cluster also exhibited a number of clinical traits previously associated with poor prognosis: namely, age (younger), grade (higher), NPI score (higher), and Basal, Her2 and TNBC proportions (all higher; Fig. 2c).

### Local production of serotonin is a major metabolic feature within the poor prognosis cluster

Having identified a cluster of personalized metabolic landscapes with significant association to poor patient prognosis, we next examined metabolic features of this emergent poor prognosis phenotype. Using the discovery dataset samples, we compared the activity of reactions in the personalized metabolic landscapes obtained for all patients within the poor prognosis cluster (134 patient samples) with all other tumours (863 patient samples). This approach identified 632 Differentially Activated Reactions (DARs) with an adjusted p-value < 0.05 (Table S2). These DARs can be further sub-divided into sets with identical mean activities within the poor prognosis group and the remaining samples (indicated by colored blocks in Table S3). To further explore this sub-division, we examined the activities on a sample-by-sample basis, confirming that within each set the DARs have identical activity across all samples within either the poor prognosis samples or all other samples. Thus, these sets of DARs represent metabolic pathways operating differentially between the poor prognosis samples and all other samples, which we will refer to as Differentially Activated Pathways (DAPs). We note that these DAPs reflect the emergence of metabolic flux routes resulting from the interplay between the global gene expression program and the stoichiometry of a whole genome scale metabolic network, rather than standard metabolic pathway map names (e.g. KEGG) assigned to differentially expressed metabolic genes.

The three DAPs most significantly divergent between the poor prognosis samples and all other samples are listed in table 1. The most statistically significant of these was degradation of casomorphins: 106/134 (79%; 95% CI 71–85%) of the poor prognosis cluster patient samples were predicted to carry flux through the casomorphin degradation pathway, compared to only 96/863 (11%; 95% CI 9–13%) in the remaining discovery set patient samples. To further examine this DAP, we examined the expression of the transcript encoding the rate-limiting reaction in casomorphin degradation, angiotensin converting enzyme 2^15^,^16^, which was significantly over-expressed in the poor patient prognosis cluster compared to all other tumours (adjusted p value = 2.5e-61).

Enhanced degradation of casomorphin would lead to an increase in N-acetyl-seryl-aspartyl-lysyl-proline peptides, which are casomorphin degradation products. It is thus pleasing to note that the second DAP identified in Table 1 represents N-acetyl-seryl-aspartyl-lysyl-proline transport reactions: 108/134 (80%; 95% CI 73–96%) of the poor prognosis cluster patient samples were predicted to exhibit increased N-acetyl-seryl-aspartyl-lysyl-proline transport, compared to only 186/863 (22%; 95% CI 19–24%) in the remaining discovery set patient samples.

**Figure 3:**
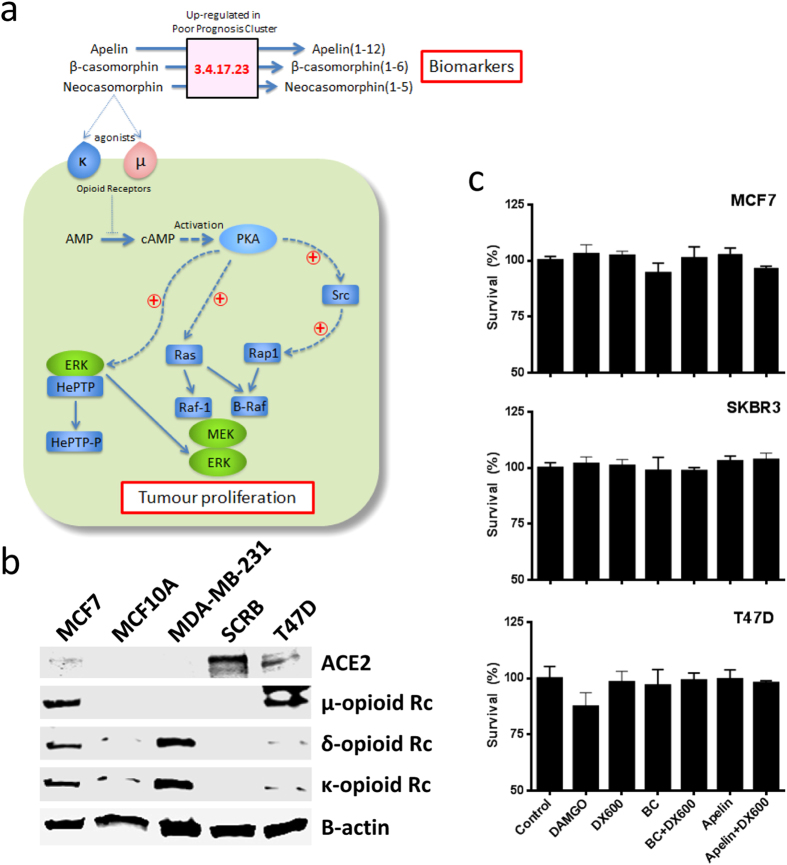
Deregulated casomorphin metabolism is a metabolic feature of poor patient prognosis tumours but is not associated with enhanced cell viability *in vitro*. (**a**) Personalized GSMNs were derived from the 997 patient transcriptome profiles within the Metabric discovery set. Comparison of the 134 personalized GSMNs associated with poor patient with the remaining 863 personalized reveals casomorphin degradation as a major driver for this separation, through increased ACE2 expression (E.C. 3.4.17.23, indicated in red). Exposure of breast cancer cell lines with the ACE2 inhibitor DX600, or β-casomorphin had no significant impact on cell proliferation. (**b**) Expression of network components (ACE2, MOR, DOR and KOR) in breast cancer cell lines. (**c**) Pharmacological perturbation of proposed network for 72h in MCF7, SKBR3 and T47D cell lines has not significant impact on the cell proliferation rate. Results are expressed as a percentage of vehicle control; each data point represents the mean of a minimum of three independent experiments of 3 wells per experiment, with error bars representing the standard error of the mean (SEM).

As casomorphins act as opioid peptides it has been suggested that they exhibit anti-proliferative and anti-tumorigenic properties^17^,^18^,^19^, most likely through decreased PKA-mediated effects on ERK signaling pathways^20^ (Fig. 3a). However, such findings have been controversial, with more recent work suggesting this link is context-dependent^21^,^22^. In addition, the use of ACE inhibitors as co-therapies during breast cancer chemotherapy has been examined and found to be ineffective, further questioning the importance of this regulatory pathway in tumour development^23^. Western blot analysis revealed that MCF7, SKBR3 and T47D breast cancer cell lines express both ACE2 and opioid receptors (Fig. 3b), making them suitable models to examine this question. Breast cancer cell lines were exposed for 72 hours to 1μM of beta-casomorphin, the opioid receptor agonist DAMGO, the ERK activator apelin, or beta-casomorphin / apelin in the presence of the ACE2-specific peptide inhibitor DX600. As can be seen from Fig. 3c, none of the cell lines exhibited a positive effect on cell viability when challenged under any of these conditions; this lack of response was also confirmed using an invasion assay (data not shown). These data are consistent with the conclusion that casomorphin is not a causative agent for an increased proliferative ability within the poor prognosis group. However, as casomorphin hydrolysis products can be detected in serum, elevated levels have the potential to identify patients within the poor prognosis cluster, and may thus represent a novel prognostic stratification biomarker^24^,^25^,^26^.

Further examination of metabolic pathways differentially regulated between poor prognosis tumours and all other tumours reveals a dysregulation in serotonin production: 82/134 (61%; 95% CI 53–69%) of the poor prognosis cluster patient samples were predicted to exhibit an active serotonin production pathway, compared to only 102/863 (12%; 95% CI 9–14%) in the remaining discovery set patient samples. This is due to the active state of DOPA decarboxylase (DDC; Recon 2 name: 3-Hydroxy-L-tyrosine carboxy-lyase)-mediated reactions in all poor prognosis sample-specific GSMNs, the final step in the conversion of tryptophan to serotonin. Up-regulation of DDC was confirmed at the transcript level within the Metabric discovery dataset (p-value < 0.001).

**Figure 4:**
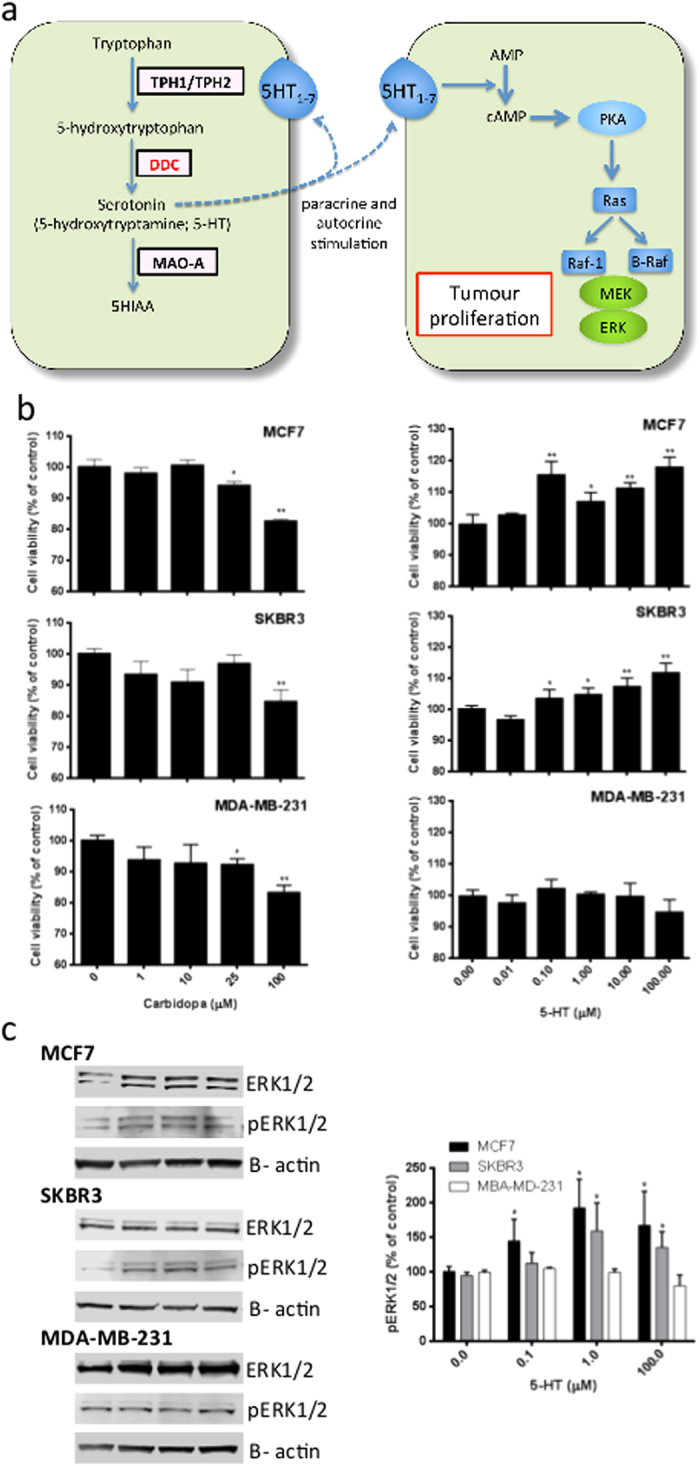
Active serotonin production is a key metabolic feature of poor patient prognosis tumours and is associated with enhanced cell viability in vitro. (**a**) Personalized GSMNs were derived from the 997 patient transcriptome profiles within the Metabric discovery set. Comparison of the 134 personalized GSMNs associated with poor patient with the remaining 863 personalized reveals an increased flux toward serotonin production (up-regulated reactions in red). MCF7, SKBR3 and MDA-MB-231 cells were exposed to varying concentrations of serotonin or the DDC inhibitor carbidopa, as indicated, for 72 hours and (**b**) proliferation measured by MTT assay, or (**c**) ERK1/2 phosphorylation measured by Western blotting. Results are expressed as a percentage of vehicle control; each data point represents the mean of a minimum of three independent experiments of 3 wells per experiment, with error bars representing the standard error of the mean (SEM). *p < 0.05, **p < 0.01 by one-way ANOVA with Dunnett’s multiple comparison correction.

In breast tissue development, serotonin acts as an autocrine-paracrine regulator^27^, with production at both the local and systemic level. Increased plasma serotonin has been suggested as a poor prognosis biomarker for a number of cancers, including intestinal, prostate and breast^28^,^29^,^30^. This is commonly thought to be due to systemic increases in serotonin production, mainly derived from the intestine and platelets, but here we focus on the importance of local production within the tumour microenvironment. To experimentally validate this prediction, we examined the effect of serotonin and carbidopa, an inhibitor of DOPA Decarboxylase, on breast cancer cell lines. The three cell lines examined (MCF-7, SKBR3 and MDA-MB-231) represent different tumour phenotypes: MCF-7 are estrogen receptor (ER), progesterone receptor (PR), epidermal growth factor (HER2) positive and considered mildly invasive; SKBR3 are ER/PR negative but HER2 over-expressing and possess an invasive phenotype ; MDA-MB-231 represent a triple negative, highly invasive phenotype^31^. All three cell lines express TPH1/2 and DDC, the enzymes responsible for serotonin production, plus a range of serotonin receptors (Supplementary Figure S1). As shown in Fig. 4b, exposure of all three cell lines to carbidopa elicited a concentration-dependent decrease in cell viability that was statistically significant at 25μM, consistent with previously reported IC50 values^32^. Serotonin produced a concentration-dependent increase in cell viability in both MCF-7 and SKBR3 cell lines, consistent with previous reports on the proliferative effects of serotonin on a range of cell lines (Fig. 4b^33–35^). However, no effect of serotonin was observed on MDA-MD-231 cells. Importantly, the concentrations used herein are within previously reported physiological ranges^36^, and hence are consistent with the hypothesis that local production of serotonin in tumours could have a positive effect on tumour cell viability. A putative molecular mechanism by which serotonin increases cell viabilty is shown in Fig. 4a: briefly, serotonin, acting both in a paracrine and autocrine fashion, activates 5HT receptors on tumour cells; this activation leads to an increased production of cAMP, and subsequent activation of PKA^37^,^38^; the subsequent signaling cascade ultimately activates the pro-mitogenic ERK1/2 proteins through phosphorylation^39^. To examine this potential mechanism, we have measured the phosphorylation status of ERK1/2 in MCF-7, SKBR3 and MDA-MB-231 cells following exposure to physiologically-relevant concentrations of serotonin. Figure 4c demonstrates that in the two cell lines that showed an increase in cell viability to serotonin, MCF-7 and SKBR3, a statistically significant increase in pERK1/2 was observed. In contrast, MDA-MB-231 cells demonstrate neither an increase in cell viability following exposure to serotonin (Fig. 4b),nor an increase in ERK1/2 phosphorylation (Fig. 4c).

To relate the concentrations of serotonin used in these experiments to those produced by the cells under basal conditions, we measured serotonin levels in conditioned medium by ELISA. In medium conditioned by MCF7, SKBR3 or MDA-MB-231 cells, the levels of serotonin were 14.2 ± 1.4nM, 14.1 ± 1.2nM, and 14.7 ± 1.4nM, respectively. Addition of serotonin to the medium caused the expected increase in medium levels, while addition of 25μM carbidopa for 72 hours had no significant effect on serotonin levels in conditioned medium. The latter is perhaps not unsurprising given the large excess of medium used *in vitro*, being approximately 2000-fold larger than the cellular volume. More likely, any changes in serotonin concentration would only be observed within the (relatively) small and slowly diffusing liquid-membrane interface.

While we present a potential molecular mechanism underlying our observed effects, it should be noted that this does not exclude other potential mechanisms. Indeed, the discord between the effects of carbidopa and serotonin in MDA-MB-231 cells would support the existence of at least one alternate mechanism. For example, an indirect effect on cell viability may exist through altered tryptophan metabolism, which is important in NAD biosynthesis, and may hence impact on mitochondrial functioning^40^. Alternatively, other metabolites produced by DDC, such as dopamine, may play a role. Further experimental work, including genetic knockdown of individual components, would be required to fully describe all potential molecular mechanisms. However, the data presented shows a strong association between active serotonin production, ERK activation and increased cell viability. In addition, we note that reports in the literature on the response of MDA-MB-231 cells to serotonin are inconclusive, demonstrating both proliferation and toxicity in a concentration-dependent manner. Our data are not inconsistent with these published reports. We conclude that MDA-MB-231 cells represent an aggressive phenotype, but one that does not reflect the phenotype of the tumours present within the poor prognosis cluster. Indeed, this reflects the widely held view that *in vitro* cell lines are often poorly reflective of the *in vivo* situation that they are trying to model. Rather, we demonstrate that MCF-7 and SKBR3 may represent mechanistically validated models for examining the biology of this poor prognosis cluster.

Serotonin supply to tumour cells may occur at the local (i.e. intratumor) or remote (i.e. systemic) levels, with platelets and the intestine major sources of serotonin within the body^41^. As such, any effect on tumour growth is likely to be a result of the combined impact of local and circulating systemic serotonin, with increased plasma serotonin previously linked to cancer progression^28^,^29^,^30^.

We thus provide an important mechanistic underpinning for the growing body of evidence linking serotonin production to cancer progression^34^. Our data predicts that a subset of patients exhibit active serotonin production within the tumour microenvironment, and we demonstrate that serotonin has a positive impact on cell viability of breast cancer cell lines *in vitro*. However, further work is required to demonstrate any causal relationship between local serotonin production *in vivo* and tumour proliferation and/or patient prognosis. Our work also supports the concerns surrounding the administration of serotonin-specific re-uptake inhibitors (SSRIs) in cancer patients^42^,^43^,^44^,^45^. The poor prognosis cluster identified in this work has active local production of serotonin and could make these patients exquisitely sensitive to further increases in serotonin caused by concomitant treatment with SSRIs. As well as motivating further clinical work in this area, our findings provide a potential explanation to the mixed epidemiological data, with only a sub-set of breast tumours possessing the molecular landscape required to overproduce serotonin and thus make them sensitive to the effects of SSRIs.

### Tumours within the poor prognosis cluster exhibit active extracellular matrix and membrane remodeling reactions

Having experimentally investigated a major metabolic pathway whose activity is associated with poor patient prognosis, we further examined the remaining DAPs. Only DAPs containing 10 or more reactions were considered, as these represent the major metabolic alterations evidenced by both gene expression changes and stoichiometric coupling into sets of reactions having the same activity in all samples. Four DAPs, consisting of 22, 54, 103 and 30 reactions (Table S3), contain metabolic functions consistent with Extracellular Matrix (ECM) and membrane remodeling during tumour invasion. After the serotonin DAP, the next most significant DAP is a 22 reaction DAP representing metabolism of chondroitin sulphate, a component of the ECM. This DAP is active in the poor prognosis cluster compared to all other tumours (adjusted p-value < 1.25e16), implying increased degradation of chondroitin-, heparin- and keratin sulphates, which has been shown by numerous studies to play a key role in tumour progression through extracellular matrix remodeling^46^,^47^. As proteoglycans such as chondroitin sulphate can be detected in the blood^48^, our work supports their development as prognostic biomarkers^46^. It should be noted that while our work supports the use of proteoglycans as prognostic biomarkers for identification of our poor patient prognosis cluster there may still exist heterogeneity within this stratified cluster. For example, our analysis predicts that while there are significantly more individuals with active chondroitin sulphate metabolism in the poor prognosis group compared to all other clusters, the distinction is not absolute: it is likely that only a panel of stratification biomarkers would fully stratify patients according to their prognosis^49^. Likewise, while we present several attributes of the poor prognosis cluster, we cannot definitively state that they would all exist in every individual within the cluster.

In addition to active metabolism of chondroitin sulphate in the poor prognosis cluster, we also observe alterations in the synthesis of cell surface glycans, important determinants of cell-cell adhesion. Three DAPs, consisting of 45, 103 and 30 reactions, are statistically active in the poor prognosis cluster tumours compared to all other tumours. The largest of these DAPs (103 reactions; adjusted p-value < 2.3e-4) contains reaction stoichiometry dependent on N-acetylglucosaminyltransferase, a key enzymatic activity in the synthesis of multi-antennary chains within the Golgi, which have been shown to promote cell detachment and invasion^50^. Likewise the 54 and 30 reactions DAPs (adjusted p-values p < 7.9e-5, and p < 3.9e-3, respectively) are involved in lysosomal processing of sialic acid glycoconjugates and N-acetylglucosaminylphosphatidylinositol in the endoplasmatic reticulum and predictive of increased remodelling of cell surface glycans.

In summary, we conclude that the poor prognosis cluster represents primary tumours in which metabolism has been already transcriptionally reprogrammed towards remodeling of the surrounding ECM and cell surface glycans. These tumours are poised for invasion and are highly likely to become metastatic, consistent with the poor patient survival observed in the clinical follow-up data.

### Incorporation of metabolic network knowledge leads to discovery of metabolic features that are not discovered by analysis of transcriptome data alone

In this work we have for the first time used knowledge about the stoichiometry of human cellular metabolism to constrain analysis of the Metabric transcriptome dataset. To evaluate the added value of this approach we have compared our results with analysis of transcriptome data alone. First, we note that association of increased serotonin biosynthesis with poor prognosis was not reported in original Metabric publication, where transcriptome data was analyzed by approaches based on overrepresentation of formal gene function descriptors in the lists of differentially expressed genes. While DDC was detected as a differentially expressed gene, this alone did not result in detection of deregulated serotonin synthesis as an important feature of poor prognosis tumours. Only through the incorporation of the GSMN constraints in our analysis were 21 further reactions stoichiometrically coupled to DDC, leading to the emergence of this behaviour in our analysis. A reason that traditional over-representation approaches fail to identify this important emergent behavior is that all other enzymes in this pathway are constitutively expressed. As such, no over-representation of pathway components exists, but the presence of all 22 coupled reactions within sample-specific GSMNs for the poor prognosis patients is able to have a significant impact on flux through the pathway.

We also note that we discover DAPs that represent a set of reactions operating in the context of all other reactions in the metabolic landscape derived by analysis of whole-genome transcriptome data in the context of the GSMN. This includes transport of extracellular substances, reflecting nutritional requirements of the tumour. Thus our DAPs differ from static pathway descriptors such as KEGG pathway names, which are frequently assigned to individual differentially expressed genes even if a pathway cannot actually operate due to, for example, down-regulation of metabolic genes in other parts of the network preventing synthesis of pathway precursors. To confirm that our results could not be obtained without the context (constraints) of the entire GSMN model, we classified tumours in the discovery and validation datasets using the absence/presence calls of metabolic genes alone. This analysis already used Recon 2 information to extract a subset of metabolic genes, but mechanistic modeling of network context was not performed. As shown in Supplementary Figure S2, we could not discover any cluster significantly associated with poor prognosis by this method. Hence, a whole-cell metabolic network context (i.e. the constraints of the entire GSMN model) was key for the discovery of the metabolic features of poor prognosis tumours, and the generation of mechanistic hypotheses for experimental analysis.

To further demonstrate the merits of such integrative analysis we also undertook a standard statistical over-representation analysis of the transcriptomic data to examine if the metabolic features associated with the poor prognosis cluster could be identified through traditional approaches. For this analysis we used DAVID, a well-established resource for functional gene annotation and enrichment analysis^51^. The top 1000 differentially expressed genes and the list of statistically overrepresented molecular process descriptors are presented as supplementary Table S4. It is important to note that these differentially expressed genes are based upon the poor patient prognosis cluster identified through metabolic landscape reconstruction and hence represent a best-case scenario for statistical over-representation analysis to identify similar molecular signatures to those found through DAP analysis. The metabolic features of the poor prognosis cluster, including the experimentally validated increase in serotonin synthesis were not discovered as significantly over-represented functional descriptors. While this demonstrates the added value of analysis incorporating network constraints, we would recommend such an approach as complimentary to traditional approaches. For example, the top over-represented DEG terms within the poor prognosis group include activation of cell division. As the GSMN includes only metabolic and transport reactions, such an analysis would not indicate cell cycle pathways as they are not included. Thus, the over-representation of cell cycle regulation processes motivates construction of analysis protocols integrating stoichiometric models, allowing further insight into the biological mechanisms that underpin this phenotype.

Incorporation of mechanistic network knowledge in analysis of -omics data has been previously shown to be a successful approach^6^,^8^,^52^. The first analysis of the Metabric dataset reported here further demonstrates applicability of this approach to generate experimental leads in the investigation of molecular mechanisms operating in diseased tissue.

## Discussion

We have analysed the Metabric dataset in the context of the GSMN Recon 2, integrating for the first time the largest functional genomics study on breast cancer tumours with the most comprehensive mechanistic model of human biochemistry created to date. We have generated personalized metabolic landscape for all samples included in the Metabric dataset. Clustering of these personalised metabolic landscapes and capitalisation on the linked clinical follow-up information allows discovery of a patient group, confirmed in the independent validation dataset, where global metabolic activity is associated with poor prognosis. In addition, the incorporation of thousands of metabolic network constraints into the analysis of transcriptome data of an unprecedented number of samples highlighted DAPs: sets of stoichiometrically coupled reactions representing differentially regulated pathways that determine the emergent poor prognosis phenotype for these tumours. Experimental investigation of the two top DAPs highlighted active serotonin biosynthesis as a potential molecular mechanism associated with poor patient prognosis. Combined with DAPs predicting ECM and membrane remodelling, this predicts a metabolic landscape consistent with a highly proliferative and invasive tumour, consistent with the poor survival of patients within this cluster.

One clinical implication of the association between active local serotonin production and poor patient prognosis is the potential for a patient group where administration of the SSRI class of anti-depressants may have implications for tumour growth and invasion, potentially extending any serotonin-dependent increases in tumour cell viability. Epidemiological evidence on the safety of SSRI use in breast cancer patients is inconclusive, with studies showing both positive and negative risk:benefit ratios^42^,^43^,^44^,^45^. Our findings suggest that one reason for this lack of clear epidemiological evidence is that only a sub-set of breast tumours possess the metabolic landscape required to actively produce serotonin and thus be sensitive to further increases caused by SSRI use. This study motivates further work in this area to fully describe any causal relationship between local serotonin production and tumour growth *in vivo*. Moreover, our metabolic landscape suggests that casomorphin or chondroitin sulphate degradation products could represent prognostic biomarkers^46^ for stratification of patients into this poor prognosis cluster, and for whom the use of SSRIs could be contra-indicated.

In summary, our analysis of the Metabric dataset in the context of GSMN Recon 2 reveals a poor prognosis group with differentially activated pathways such as serotonin production that underlie this aggressive tumour phenotype. Our result encourages the incorporation of mechanistic network information into the analysis of other clinical functional genomics datasets.

## Methods

We used gene expression data from the discovery set of 997 tumour samples and validation set of 995 tumour samples generated by the Metabric consortium. The data were obtained from from the European genome-phenome archive (https://www.ebi.ac.uk/ega/home)^2^ following approval by Data Access Committee. The Recon 2 GSMN, which is the most comprehensive reconstruction of human metabolism, encompassing 5,063 metabolites interconverted through 7,440 unique reactions was acquired in SBML format from its repository database http://humanmetabolism.org11.

### Generation of Personalised Metabolic Landscapes

An overview of the analysis pipeline is provided in Fig. 1, with comprehensive details available as supplemental material. For each tumour, transcriptomic data was first pre-processed following the protocols of the original Metabric publication^2^, and then presence or absence of a transcript determined from the Illumina detection calls (p < 0.01). The gene-reaction association rules from the Recon 2 genome-scale metabolic network model were then used to calculate reaction states as −1 (absent) or 0 (present) based on presence/absence calls for each tumor under investigation. Out of 1773 metabolic enzyme transcripts included in the Recon2 model, 1499 (84.5%) were assigned a presence/absence state. The Illumina platform used in Metabric publication did not include probes for the remaining 234 metabolic enzyme transcripts, and hence their activity state could not be assigned. We then used the well-established congruency approach^53^ to search for tumor-specific GSMNs maximally congruent with reaction classification based on Metabric transcriptome data while satisfying stoichiometric and thermodynamic constraints of original Recon 2 model. The congruency was calculated as the number of reactions classified as absent, and for which steady state metabolic flux equals zero in the GSMN model. The best possible solution, where fluxes of all reactions absent according to transcriptome data were simply constrained to zero violated stoichiometric and thermodynamic constraints. Thus, combinatorial optimisation algorithm was employed to identify metabolic models maximally, rather than completely consistent with transcriptome based reaction classification. The congruency approach requires discretized data on gene expression levels, and a potential issue is the use of arbitrary thresholds for discretization of over-expression. To address this, we used only two categories (present or absent), which can be robustly determined through the Illumina statistics for transcript absence. We believe that this strong evidence for absence of expression provides a more robust and valuable constraint compared to over-expression based on arbitrary thresholds.

The standard iMAT algorithm^53^ used for congruency approach applies Mixed Integer Linear Programming to determine the maximal congruency between transcriptome data and GSMN. Since there are potentially many alternate GSMNs that are maximally congruent with the transcriptome data, iMAT determines for each individual reaction the range of activities consistent with maximal solution. This requires execution of two MILP optimizations for every reaction, which we have found is too computationally expensive for generation of the large number of metabolic landscapes produced in this study. We have therefore developed Gene Expression Based Reaction Activity (GEBRA), our own variant of the standard approach. Here, we perform only one MILP optimization for each transcriptome sample-specific GSMN, Subsequently, we constrain fluxes of reactions associated with absent transcripts to the fluxes obtained in the MILP solution, and then determine fluxes of all remaining reactions by standard Flux Variability Analysis using Linear Programming. The FVA result is then used to generate a metabolic landscape by assigning non-active or active state to each of the reactions. Reactions for which both the minimal and maximal FVA flux are 0, are considered to be non-active and assigned a state of  −1, with the remaining reactions declared active and assigned the state 0. We decided not to classify active reactions further, into those which have to be active (flux range do not contain zero) and those which are undetermined i.e. can, but do not have to be active (flux range includes 0). To robustly determine reactions, which become active in particular tumour, we would have to discretize transcriptome data in a way, which introduces “upregulated” gene category. This would require an arbitrary threshold. Instead, we used more robust detection p-value to find genes, which are not expressed and use this information to identify metabolic reactions, which do not operate in particular tumour. Finally, metabolic landscape of a particular transcriptome sample is defined as an assignment of states to all Recon 2 reactions 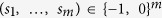 where and m is the number of metabolic reactions in Recon2.

To assess the gain of computational efficiency and any loss of accuracy due to simplification of the iMAT algorithm, we have compared reaction classification obtained by iMAT and GEBRA for transcriptome data from the NCI-H23 cell line (part of NCI-60 collection). The GEBRA approach was 80-times faster than the original iMAT method and reproduced iMAT’s results with 92% accuracy (p-value for obtaining equal number of concordant reactions by chance 4.3 × 10^−20^). Furthermore, we randomly selected 5 samples from the poor prognosis cluster and 5 samples from remaining part of the discovery dataset. We then generated metabolic landscapes for these samples using both the iMAT and GEBRA approaches, and determined DARs. There was a 90% agreement in the DARs determined by the two methods, and both methods discovered the serotonin and casomorphin DARs presented in results section. Thus GEBRA and iMAT analyses lead to the same biological insight, but iMAT is computationally too expensive to generate 2000 metabolic landscapes.

Detailed formulation of the Fast iMAT algorithm and comparison with the standard approach is given in the Supplementary Material and Supplementary Table S5. The algorithm has been implemented in our SurreyFBA software (v2)^54^, which is available at http://sysbio3.fhms.surrey.ac.uk/sfba/index.html. The JyMet interface of SurreyFBA was used for conversion of Recon 2 SBML to SurreyFBA format. GEBRA calculations were undertaken through a command line solver of SurreyFBA2 used within a computational pipeline implemented in python and R Bioconductor^55^.

### Cluster analysis, Kaplan-Maier curves and identification of Differentially Activated Reactions

Metabolic landscapes were classified by the k-means approach using the R-package clValid. The input data for clustering of discovery dataset consisted of 997 metabolic landscapes (cases) characterized by 3016 reactions states (variables): reactions with the same activity in all tumours were not included as variables as they provide no useful information: This resulted in clustering being undertaken using 3016 and 3471 reactions for the discovery and validation datasets, respectively. A range of clusters from 5 to 10 was investigated, consistent with the 10 clusters previously identified from transcriptome analysis in the original Metabric publication. For each of the clusters, clinical follow-up data were used to calculate Kaplan-Maier curves, plus the associated p-value, with the R-package survival. Classification into 8 clusters yielded the lowest p-values and was used for further analysis.

Differentially Affected Reactions (DARs) were identified as those that had significantly different activity in the poor prognosis cluster compared to the remaining samples. Given the very large number of samples, we compared mean reaction activities between groups of samples using a t-test with Bonferoni correction for multiple comparisons. Reactions with adjusted p-values < 0.05 were considered to be DARs, while sets of DARs that had identical activities in all samples were considered as Differentially Active Pathways (DAPs).

### Cell Culture

MCF-7, MCF10A, MDA-MB-231, SKRB and T47D cell lines were purchased from ECACC. All cells were grown in Dulbecco’s modified eagle medium (DMEM) with phenol red and L-glutamine, and supplemented with 10% Fetal bovine serum (FBS) and, 100 units/ml penicillin, 0.1mg/ml streptomycin sulphate, and 0.25μg/ml amphotericin B.

### Cell viability assay

Cell viability was assessed by the 3-(4,5-dimethylthiazol-2-yl)2,5-diphenyl tetrazolium bromide (MTT) assay: treated cells and controls were incubated with 0.5 mg/mL MTT for 2½ h and the resultant formazan salt dissolved in DMSO and its absorbance measured at 540 nm. Results are expressed as a percentage of vehicle control; each data point represents the mean of a minimum of three independent experiments of 3 wells per experiment, with error bars representing the standard error of the mean (SEM). Statistical significance was examined through a one-way ANOVA with Tukey’s multiple comparison correction, using GraphPad Prism (v6, GraphPad Software Inc., La Jolla, USA).

### Protein analysis

Total protein extracts were resolved on 8–16% SDS-polyacrylamide gels and then transferred electrophoretically to Hybond ECL nitrocellulose membranes (Amersham Biosciences, Little Chalfont, Bucks, UK). Membranes were blocked (1 hour) in 5% fat free dried milk and then probed with primary antibodies against ACE2 (ab15348), MOR (ab17934), DOR (ab176324), or KOR (ab137264) (all 1:1000) for one hour, followed by anti-rabbit (1:10000) or anti-mouse IgG (1:10000) IRDye 800 CW secondary, as appropriate, for one hour at room temperature. The membrane was then imaged using an Odyssey Family Imaging System (LI-COR Biosciences). Immunoblots were also probed with an antibody against β-actin (ab-8226; 1:1000) for one hour, followed by anti-mouse IgG IRDye 800 CW (1:10000) for one hour to ensure even loading per lane. All antibodies were purchased from Abcam (Cambridge, UK) or Licor (Cambridge, UK), as appropriate.

## Additional Information

**How to cite this article**: Leoncikas, V. *et al.* Generation of 2,000 breast cancer metabolic landscapes reveals a poor prognosis group with active serotonin production. *Sci. Rep.* **6**, 19771; doi: 10.1038/srep19771 (2016).

## Supplementary Material

Supplementary Information

Supplementary Table S1

Supplementary Table S2

Supplementary Table S3

Supplementary Table S4

Supplementary Table S5

## Figures and Tables

**Table 1:** Top three groups of Differentially Active Reactions.

Recon 2 reaction names	Samples with active reaction (95% CI)			
	Poor prognosis	All other samples	Adjusted p-value	Pathway
beta-casomorphin exchange, neocasomorphin exchange, apelin-13 exchange, beta-casomorphin (1–6) exchange, neocasomorphin (1–5) exchange, apelin (1–12) exchange, RE0936, RE0937, RE0938	79% (71–85%)	11% (9–13%)	2.04E-70	Casomorphin degradation
N-acetyl-seryl-aspartyl-lysyl-proline exchange, N-acetyl-seryl-aspartate exchange, lysyl-proline exchange, RE2445	81% (74–88%)	21% (17–25%)	7.91E-44	Uptake of casomorphin degradation products
3-Hydroxy-L-tyrosine carboxy-lyase, 4-Hydroxyphenylacetaldehyde:NADP + oxidoreductase, 5-Hydroxy-L-tryptophan decarboxy-lyase, 4-Hydroxyphenylacetate exchange, Dopamine exchange, alpha-N-Phenylacetyl-L-glutamine exchange, Serotonin exchange, Tyramine O-sulfate exchange, hydroxyphenylacetate transport via diffusion, phenylacetate-CoA ligase, Phenethylamine oxidase, Phenylacetyl-CoA:L-glutamine alpha-N-phenylacetyltransferase, PHEACGLN extracellular transport via diffusion, L-Phenylalanine carboxy-lyase, Tyramine O-sulfate transport (diffusion), Tyramine Sulfotransferase, L-Tyrosine carboxy-lyase, Tyramine:oxygen oxidoreductase(deaminating), 3,4-Dihydroxy-L-phenylalanine exchange	61% (52–69%)	12% (9–15%)	3.79E-42	Serotonin synthesis

Table shows top 47 DARs from Table S1 sorted by adjusted p-value. Each row shows group of reactions with identical activities across all Metabric samples in discovery set. The percentage of samples in which reactions were active in poor prognosis cluster and all other samples are given. The adjusted p-value for a difference between mean reaction activities in poor prognosis cluster and remaining samples is calculated as described in Methods.
