# Supplementary material for: Generation of 2,000 breast cancer metabolic landscapes reveals a poor prognosis group with active serotonin production
Source: Sci Rep. 2016 Jan 27;6:19771. doi: 10.1038/srep19771 (PMC4728432; doi:10.1038/srep19771)
Supplement: Supplementary Information [file srep19771-s1.pdf]

SUPPLEMENTARY INFORMATION

Generation of 2,000 breast cancer metabolic landscapes reveals a poor prognosis group with active serotonin production

Vytautas Leoncikas, Huihai Wu, Lara T. Ward, Andrzej M. Kierzek and Nick J. Plant

TABLE OF CONTENTS

1 MATERIALS **2**

2 METHODS **2**

2.1 Transcriptomic data pre-processing **2**

2.1.1 Data import and pre-processing **2**

2.1.2 Probe annotation **2**

2.1.3 Determination of gene expression presence/absence from pre-processed transcriptomic data **2**

2.2 Creation of personalized GSMNs using transcriptome data **3**

2.3 Clustering of personalized GSMNs **6**

2.4 Differential gene expression **7**

2.5 Statistical Analysis **7**

3 SUPPLEMENTARY FIGURES **7**

3.1 Figure S1: Breast cancer cell lines express the major components for serotonin production and a range of serotonin receptors **7**

3.1.1 Methods 7

3.1.2 Results 8

3.2 Figure S2: Identification of tumours associated with poor patient prognosis requires interpretation of Metabric transcriptomic data within the context of a genome-scale metabolic network **10**

3.2.1 Methods 10

3.2.2 Results 10

4 SUPPLEMENTARY TABLES **11**

REFERENCES **12**

1 MATERIALS

As part of the Metabric (Molecular Taxonomy of Breast Cancer International Consortium) study, transcriptomic data from over 2000 breast tumours was generated, with full details of the patient population available in the accompanying publication [1]. The data is available through the European Genome-phenome archive (https://www.ebi.ac.uk/ega/home) under study ID EGAD00010000162 – Illumina HT 12 IDATS. The microarray platform used in the Metabric study was the Illumina HumanHT 12v3 beadchip.

The human genome scale metabolic network (GSMN) *Recon 2* has been downloaded from its public repository (http://humanmetabolism.org/[2]).

2 METHODS

2.1 Transcriptomic data pre-processing

Raw transcriptomic data was first pre-processed to remove data from bad quality probes, normalize expression levels across bead arrays and summarize probeset data to derive expression levels for each target gene. All data manipulation and analysis were performed using scripts written in the R statistical programming language and the Bioconductor[3] and RStudio [4] interfaces.

2.1.1 Data import and pre-processing

Transcriptomic data within the Metabric dataset was generated using the Illumina HumanHT-12 v3 platform. The Bioconductor package *beadarray* was used to import raw images, generate quality assessment information, carry out image processing to adjust for spatial artefacts, and undertake background correction. Probes were then filtered by detection score, with a cut off p-value set as 0.01 [3,5]

2.1.2 Probe annotation

The Bioconductor package *illuminaHumanv3.db* was used to provide a mapping between the Illumina probe identifiers and common gene symbol identifiers [5].

2.1.3 Determination of gene expression presence/absence from pre-processed transcriptomic data

Following pre-processing, transcript levels were classified as absent or present based on detection calls. This classification removes the potential bias associated with the arbitrary

thresholds required for setting discrete gene expression states (down-regulated, unaffected, up-regulated).

We note that each transcript can be detected by multiple probe sets, and one probe set can identify more than one target gene. Hence, it is necessary to weight presence/absence calls against the specificity of the probesets. Genes were considered to be present if half or more probes for a given probeset were detected. Probes detected as present were assigned a value of 0; absent probes were assigned value of -1. If the mean for all probes in a given probset was higher than -0.5 the gene was considered expressed and not expressed otherwise.

2.2 Creation of personalized GSMNs using transcriptome data

The presence/absence calls for the expression of each gene on the Illumina beadarray were analysed in the context of Recon2 Genome Scale Metabolic Network (GSMN) to derive personalised metabolic landscapes for each sample. Recon2 is the most comprehensive reconstruction of global human metabolism, encompassing 5,063 metabolites interconverted through 7,440 unique reactions [2]. The list of metabolic reaction formulas is derived from the repertoire of metabolic enzymes encoded in human genome. The dependence of a reaction on the genes encoding protein subunits of an enzyme catalysing this reaction is expressed as a Boolean gene-reaction association rule. Here, we have downloaded a Systems Biology Markup Language (SBML) file from http://humanmetabolism.org/, which defines metabolites, reactions and gene-reaction association rules. The SBML file has been imported into version 2 of our SurreyFBA software, which was subsequently used for all simulations described below.

We used a modified version of the iMAT method of Shlomi and colleagues [6] to integrate transcriptomic presence/absence call data with Recon 2 model and derive metabolic landscapes. The iMAT uses well-established Constrained Based Modelling (CBM) approach, where whole-cell metabolic model is simulated at steady state. The variables of the model are reaction fluxes rather than metabolic concentrations. The metabolic reaction formulas are used to create stoichiometric constraints, where for each metabolite the sum of fluxes producing and consuming this metabolite equals 0. Additional thermodynamic constraints are created using information about reaction reversibility. Transcriptome data are then discretised into three levels: -1, 0 and 1 described as “lowly”, “moderately” and “highly” expressed genes in original publication. Discretised data are used to identify a subset of reactions in GSMN, which are metabolically active for a particular pattern of gene expression. The iMAT searches for the reaction sets, which are maximally congruent with transcriptome data in terms of following criteria: i) all stoichiometric and thermodynamic constraints are satisfied ii) maximal number of reactions associated with “lowly” expressed genes is excluded from

the result iii) maximal number of reactions associated with “highly” expressed genes is included into solution. The trivial solution including all reactions associated with highly expressed genes and excluding all reactions associated with “lowly” expressed genes usually violates stoichiometric and thermodynamic constraints. Thus, the iMAT performs Mixed Integer Liner Programming (MILP) optimisation to find a set of reactions satisfying all constraints and matching maximal number of reactions with associated gene expression status. There may be multiple reaction sets satisfying optimisation objective to the same extent. To address this problem iMAT determines how activity of each reaction affects the solution. This requires execution of two MILP optimisations for each reaction. The final result is classification of each reaction as active in all alternative solutions, inactive in all alternative solutions or undetermined. Detailed formulation of iMAT is given elsewhere and will not be re-iterated here.

The MILP is computationally expensive as a single solution requires iterative execution of multiple Linear Programming (LP) optimisations of the entire GSMN model. Since iMAT executes two MILP optimisations for each reaction, the iMAT analysis of a single transcriptome sample in the context of Recon 2 requires 14,880 MILP optimisations. We have found that iMAT is too computationally expensive to analyse each of the 2000 transcriptome samples in METABRCIK dataset. To address this issue, we have modified iMAT such that only one MILP solution per transcriptome sample is needed, thus increasing the computational efficiency. Since we use gene expression data to identify, which reactions in the GSMN are non-active we name our approach Gene Expression Based Reaction Activity (GEBRA).

The GEBRA uses discretized expression data as input. However, contrary to iMAT we use two rather than three levels and use detection calls rather than array signal (see 2.1.3 above). As argued in main manuscript this is more robust as no arbitrary threshold on array signal is required. Thus, the genes which transcripts are absent are assigned -1 and genes which transcripts are present are assigned 0. Subsequently, for each reaction i the gene-reaction association rules are used to calculate reaction state denoted by s_i. The gene names are replaced by levels from {-1, 0} and logical “and” and “or” operators are replaced with max and min operations respectively. Resulting reaction states also assume -1 or 0. We note that encoding as -1 or 0, rather than logical “true” or “false” is convenient as the resulting state s_i can be directly used as coefficient of objective functions: reactions with state -1 will contribute negatively to the objective, other reactions do not contribute at all. The GEBRA searches for stoichiometrically and thermodynamically feasible metabolic models, where the maximal number of reactions associated with absent genes is non-active.

After reaction states are determined, reactions are classified as forward (lower bound flux $v_{min} \geq 0$) denoted as v_i with state s_i where $i \in R_f$ (forward reaction set), reverse reactions (upper bound flux $v_{max} \leq 0$) denoted as v_j with state s_j where $j \in R_r$ (reverse reaction set), and reversible reactions ($v_{min} < 0$ and $v_{max} > 0$) denoted as v_k with state s_k where $k \in R_{fr}$ (reversible reaction set). Each reversible reaction is divided into a forward direction denoted v_k^+, and a reverse direction denoted v_k^-, both having the state s_k. Subsequently the MILP problem defined by the following equations is solved:

$$max_{v_i,v_j,v_k^+,v_k^-,b^+,b^-} \left(\sum_{i \in R_f}(s_i \cdot v_i) - \sum_{j \in R_r}(s_j \cdot v_j) + \sum_{k \in R_{fr}} s_k \cdot (v_k^+ + v_k^-) \right) \quad [1]$$

subject to the constraints:

$$S \times v = 0, \;\; S \in R^{n \times m}, v \in R^m \quad [2]$$

$$v_{min} \leq v \leq v_{max} \quad [3]$$

$$v_k^+ + b \cdot v_{max} \leq v_{max} \quad [4]$$

$$v_k^- + b \cdot v_{min} \leq 0 \quad [5]$$

$$b \in [0, 1] \quad [6]$$

Eqn 1 defines the objective function of the MILP problem. Eqn 2, defines stoichiometric constraints and flux balance relations at steady state, where S is a $n \times m$ stoichiometric matrix with n metabolites and m reactions and v is a vector of m reaction fluxes. Eqn 3 defines flux bounds that express reaction reversibility (thermodynamic constraints) and boundary conditions i.e. the set of active extracellular nutrient transporters. Here, we used set of nutrients defined in Recon 2 model and set Biomass reaction flux to maximal value possible in Recon 2, thus requiring that all essential cell components are synthesized. Eqns 4-6 add additional constraints and use one Boolean control variable, b, to ensure that for every forward/reverse reaction pair only one of the reactions is active at any point, thus preventing futile cycles. The solution of this MILP problem provides fluxes for all reactions. The fluxes of reactions with state s = -1 are then constrained to their values obtained in MILP solution. The range of fluxes accessible to reactions with state s = 0 is then determined by Flux Variability Analysis (FVA). Each reaction subjected to FVA becomes objective function and its minimal and maximal value is determined by two Linear Programming (LP) optmisations. The LP is much faster then MILP, thus computational cost remains acceptable. At the end of this step each reaction is assigned a flux range $[F_{min}, F_{max}]$. Reactions with state = 0 are assigned FVA ranges, reactions with state -1 have minimal and maximal flux equal to MILP solution. The final output of GEBRA is reaction activity determined by classification of flux ranges. If $F_{min} = F_{max} = 0$ then the reaction is predicted to be inactive and assigned activity of -1; otherwise, the reaction is predicted undetermined and assigned activity of 0. The final

solution, which we call a metabolic landscape is vector a of m elements, where a_i is activity of ith reaction, either -1 (non-active) or 0 (undetermined). In other words, we determine a set of inactive reactions, such that as many reactions associated with absent transcripts (s = -1) are declared to be non-active (a = -1) as it is possible without violation of GSMN constraints.

Our GEBRA method is based on the "congruency" principle of iMAT, but uses results of single MILP solution instead of two MILP solutions for each reaction. In order to evaluate to what extent this assumption affects assignment of non-active reactions we compared GEBRA with iMAT by analysis of the transcriptome data for NCI-H23 cell line derived from a non-small cell lung carcinoma [7],[8]. To enable comparison we used only two gene expression levels ("low", "medium") in iMAT. Full details of the comparison are presented as supplementary table S5, with major points noted here. Analysis between the two approaches revealed a statistically significant overlap of 2036 reactions predicted by both GEBRA and iMAT, with a prediction accuracy of 92% (p=4.3e-20). Critically, the GEBRA approach was computationally much more efficient than the original approach. Using the same computational cluster, analysis of the NCI-H23 transcriptome took 59725 seconds using the original iMAT approach, but only 752 seconds using GEBRA; hence, GEBRA approach was nearly 80-times faster than the original method, while generating results which were nearly identical to original congruency method. The gain of computational efficiency enabled the first generation of personalised GSMNs for all 2000 tumours in METABRICK dataset and discovery of metabolic reprogramming features that were validated experimentally.

2.3 Clustering of personalized GSMNs

To identify clusters of similar personalized GSMNs for breast cancer, K-means clustering was performed using the R package *clValid* [9]. The input data for clustering consisted of 2000 GSMNs (cases) characterized by 7440 reactions states (variables). A range of clusters from 5 to 10 was investigated, consistent with the 10 clusters previously identified from transcriptome analysis in the original Metabric publication [1]. K-means clustering identified a stable, statistically significant cluster that was associated with poor patient prognosis, as determined by the cluster analysis package *fpc*. We have also performed hierarchical clustering. A statistically significant cluster of personalized GSMNs associated with poor patient prognosis was also identified by this approach. However, cluster stability was shown to be poor through bootstrap analysis.

In summary, we demonstrate that a poor prognosis cluster can be successfully recovered and is stable when K-means clustering method is used. This cluster is reproducible using a second clustering approach, although with a lower degree of robustness. Analysis of the personalized GSMNs derived from the Metabric confirmatory dataset by both K-means and hierarchical

clustering approaches identified a poor patient prognosis cluster, with this cluster being more robustly derived by K-means clustering, again.

2.4 Differential gene expression

Gene expression profiles used to derive personalized GSMNs within the poor patient prognosis cluster were compared to all other gene expression profiles within the Metabric discovery set. Differential gene expression was performed using the *beadarray* and *limma* packages[5,10], with functional clustering analysis undertaken in DAVID[11]. Statistical over-expression approach is a standard methodology to analyze transcriptomic datasets, and allows comparison between the two approaches: statistical over-representation *versus* personalized GSMN prediction

2.5 Statistical Analysis

Pairwise t-test and Wilcoxon signed-rank test was performed using GraphPad Prism (v6), and the R-packages *splines*, *survival* and *pvclust*[3,12]. Binomial probability confidence intervals were calculated using: http://www.danielsoper.com/statcalc3/calc.aspx?id=85.

3 SUPPLEMENTARY FIGURES

3.1 Figure S1: Breast cancer cell lines express the major components for serotonin production and a range of serotonin receptors

3.1.1 Methods

For in vitro analysis of DDC and Tph1/2 expression in breast cancer cell lines, SHSY5Y, MDA-MB-231, MCF7 and SKBR3 cells were cultured in 6-well plates at a density of $1x10^6$ cells per well in Dulbecco's Modified Eagle Medium with 2 mM L-glutamine, 4.5 g/L glucose, 100 units/ml penicillin, 0.1mg/ml streptomycin sulphate, 0.25µg/ml amphotericin B and 10% foetal bovine serum (FBS), at 37˚C and 5% CO2.

Western blot analysis was undertaken as previously described[13]. Briefly, total protein was extracted using RIPA buffer, and protein level quantified by the method of Lowry[14]. Thirty micrograms of total proteins was separated on precast 6-18% polyacrylamide gels, and then transferred to PVDF membrane. Membranes were blocked (1 hour) in 5 % fat free dried milk and then probed with primary antibodies against DDC (ab15348) or Tph1/2 (ab17934) for one hour, followed by anti-rabbit (1:10000) or anti-mouse IgG (1:10000) IRDye 800 CW

secondary, as appropriate, for one hour at room temperature. The membrane was then imaged using an Odyssey Family Imaging System (LI-COR Biosciences).

For in silico analysis of 5-HT receptor expression in breast cancer cell lines, the GSE12777 dataset contains gene expression profiling of 51 human breast cancer cell lines, and was downloaded from the Gene Expression Omnibus (GEO[15]). Analysis of array output files was performed within the Bioconductor R suite[3]: Data pre-processing was performed using the *affy* package[16], and gene expression levels for 24 5-HT receptors extracted.

3.1.2 Results

DOPA decarboxylase (DDC) and tryptophan hydroxylase (TPH1/2) are required for the production of serotonin from tryptohan (figure S1a). Immunoblotting demonstrates that MDA-MB-231, MCF7 and SKBR3 cells all express DDC and TPH1/2, as well as the positive control neuroblastoma cell line SHSY5Y (figure S1b). Analysis of transcriptomic data from 51 human breast cancer cell lines, including those used in the current study, demonstrates expression of multiple 5-HT receptors within the cell lines (figure S1c).

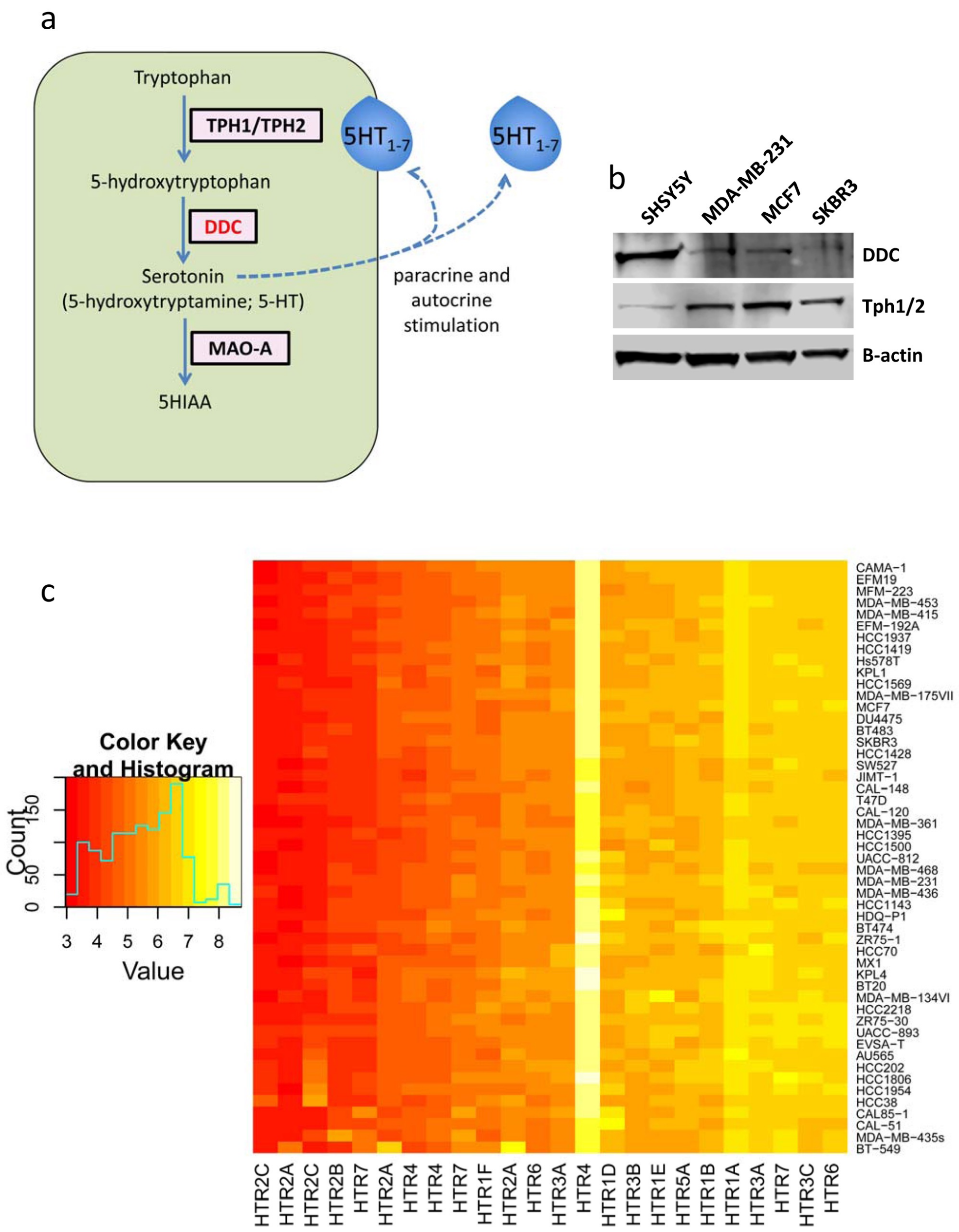

Figure S1: Breast cancer cell lines express the components for production of serotonin. (A) Representation of the serotonin production and detection system within breast cancer cells. (B) Total protein from MDA-MB-231, MDF7, and SKBR3 breast cancer cell lines, or the positive control cell line SHSY5Y (neuroblastoma-derived) was separated by SDS-PAGE. DDC and Tph1/2 protein levels were detected by Western blot; B-actin protein levels were also detected to ensure even loading. (C) Transcript levels of 27 5-HT receptors across 51 breast cancer cell lines were extracted from the GSE12777 dataset, and are presented as an expression heat map.

3.2 Figure S2: Identification of tumours associated with poor patient prognosis requires interpretation of Metabric transcriptomic data within the context of a genome-scale metabolic network

3.2.1 Methods

For each tumour within the discovery and validation Metabric datasets, the sub-set of genes that map to reactions within Recon2 was extracted. These were then classified as -1 (absent) or 0 (present) based on Illumina presence/absence calls from the Metabric transcriptomic data alone. Hence, while the GSMN was used to identify those genes that map to metabolic reaction, network connectivity was not used to generate personalised metabolic landscapes for each tumour, or for subsequent clustering. Next, k-means clustering was undertaken using a default target of eight clusters, which was previously determined to be optimal.

3.2.2 Results

Clustering of the sub-set of transcriptome data that map to metabolic genes within Recon2 could not recover any cluster significantly associated with poor prognosis for either the discovery of validation Metabric datasets. This is consistent with the assertion that a whole-cell metabolic network context (i.e. the constraints of the entire GSMN model) is key for the discovery of the metabolic features of poor prognosis tumours, and the generation of mechanistic hypotheses for experimental analysis.

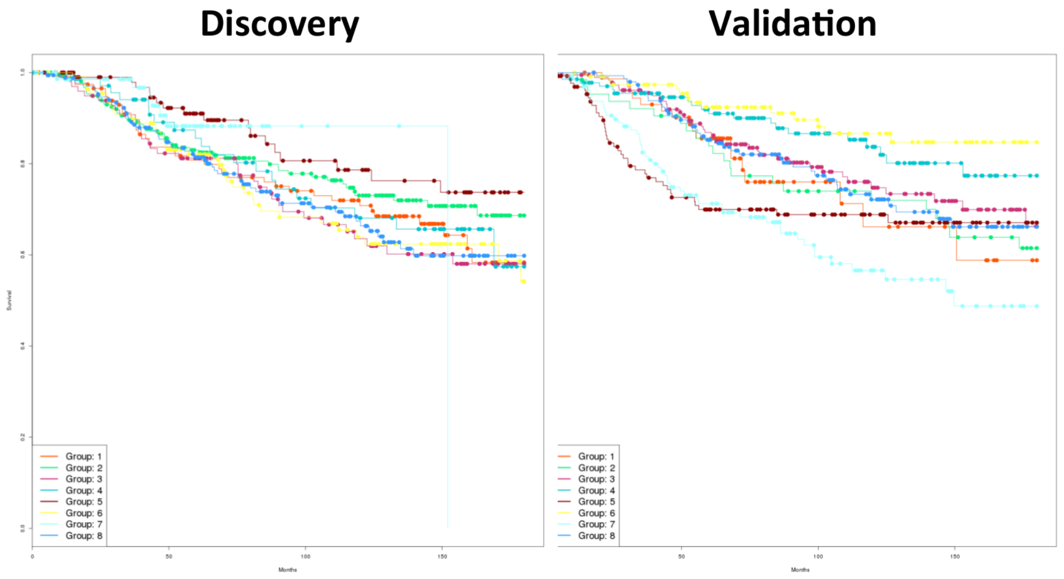

Figure S2: Clustering of tumour transcriptomes by absence/presence calls alone is insufficient to recover a cluster associated with poor patient prognosis. The sub-set of reactions mapped to Recon2 metabolic reactions was extracted from the Metabric

transcriptome data for each tumour. Genes were classified according to their Ilumina A/P call and then subject to k-means clustering

4 SUPPLEMENTARY TABLES

Table S1: Differently activated reactions for the poor prognosis clusters derived from the discovery and confirmatory datasets show 97% concordance. Personalized GSMNs were derived for breast tumours within the discovery (997 sample) and confirmatory (995) datasets, as described in methods. K-means clustering analysis reveals a cluster associated with poor patient prognosis in both datasets. To test the overlap in these two poor patient prognosis clusters, differentially activated reactions were compared using Wilcoxon signed-rank test. 2198/2273 (97%) differentially activated reactions were concordant between the discovery and confirmatory dataset-derived poor patient prognosis clusters, with only 75/2273 (3%) being different.

Table S2: Differently activated reactions between the poor prognosis cluster and all other samples within the discovery dataset. Personalized GSMNs were derived for breast tumours within the discovery (997 samples), as described in methods. K-means clustering analysis revealed a statistically significant cluster associated with poor patient prognosis. DARs between the poor patient prognosis cluster (134 samples) and all other samples (863 samples) were then identified using flux variability analysis.

Table S3: Differently activated pathways between the poor prognosis cluster and all other samples within the discovery dataset. Personalized GSMNs were derived for breast tumours within the discovery (997 samples), as described in methods. K-means clustering analysis revealed a statistically significant cluster associated with poor patient prognosis. DARs between the poor patient prognosis cluster (134 samples) and all other samples (863 samples) were then identified using flux variability analysis, and separated into DAPs based upon identical mean activities within either the poor prognosis group or the remaining samples.

Table S4: Differentially expressed genes between poor prognosis cluster and all other samples within the discovery dataset. Differentially expressed genes (DEGs) between poor prognosis cluster and all other samples within the discovery dataset were identified using the

Bioconductor package *limma*. For the top 1000 DEGs, the Illumina probe ID, gene name and gene ID are presented, along with statistical analysis supporting differential expression. DAVID Functional annotation clustering for these DEGs is presented on the second tab.

Table S5: Comparison between iMAT and GEBRA methods. Personalized GSMNs were derived from the transcriptomic data for the NCI-H23 cell line against the Recon2 GSMN by both iMAT and GEBRA methods. Raw output from each approach is provided in first tab, with a direct reaction-by-reaction of activity calls presented in the second tab. Finally, comparative statistics are provided in the third tab.

REFERENCES

1 Curtis, C. *et al.* The genomic and transcriptomic architecture of 2,000 breast tumours reveals novel subgroups. *Nature* **486**, 346-352, doi:10.1038/nature10983 (2012).

2 Thiele, I. *et al.* A community-driven global reconstruction of human metabolism. *Nature Biotechnology* **31**, 419, doi:doi:10.1038/nbt.2488 (2013).

3 Gentleman, R. C. *et al.* Bioconductor: open software development for computational biology and bioinformatics. *Genome Biol.* **5**, R80 (2004).

4 Racine, J. S. RStudio: A Platform-Independent IDE for R and Sweave. *J. Appl. Econom.* **27**, 167-172, doi:10.1002/jae.1278 (2012).

5 Dunning, M. J., Smith, M. L., Ritchie, M. E. & Tavare, S. beadarray: R classes and methods for Illumina bead-based data. *Bioinformatics* **23**, 2183-2184, doi:10.1093/bioinformatics/btm311 (2007).

6 Shlomi, T., Cabili, M. N., Herrgard, M. J., Palsson, B. O. & Ruppin, E. Network-based prediction of human tissue-specific metabolism. *Nature Biotechnology* **26**, 1003-1010, doi:10.1038/nbt.1487 (2008).

7 Gazdar, A. F. *et al.* Establishment of continuous, clonable cultures of small-cell carcinoma of lung which have amine precursor uptake and decarboxylation cell properties. *Cancer Res.* **40**, 3502-3507 (1980).

8 Grever, M. R., Schepartz, S. A. & Chabner, B. A. The National Cancer Institute: Cancer drug discovery and development program. *Seminars in Oncology* **19**, 622-638 (1992).

9 Brock, G., Datta, S., Pihur, V. & Datta, S. clValid: An R package for cluster validation. *Journal of Statistical Software* **25**, 1-22 (2008).

10 Ritchie, M. E. *et al.* limma powers differential expression analyses for RNA-sequencing and microarray studies. *Nucleic Acids Res.* **43**, e47, doi:10.1093/nar/gkv007 (2015).

11 Dennis , G. *et al.* DAVID: Database for Annotation, Visualization, and Integrated Discovery. *Genome Biol.* **4**, R60. (2003).

12 Suzuki, R. & Shimodaira, H. Pvclust: an R package for assessing the uncertainty in hierarchical clustering. *Bioinformatics* **22**, 1540-1542, doi:10.1093/bioinformatics/btl117 (2006).

13 Gee, R. H. *et al.* Inhibition of prenyltransferase activity by statins in both liver and muscle cell lines is not causative of cytotoxicity. *Toxicology* **329**, 40-48, doi:10.1016/j.tox.2015.01.005 (2015).

14 Lowry, O. H., Rosebrough, N. J., Farr, A. L. & Randall, R. J. Protein Measurement with the Folin Phenol Reagent. *J. Biol. Chem.* **193**, 265-275. (1951).

15 Barrett, T. *et al.* NCBI GEO: archive for functional genomics data sets-update. *Nucleic Acids Res.* **41**, D991-D995, doi:10.1093/nar/gks1193 (2013).
16 Gautier, L., Cope, L., Bolstad, B. M. & Irizarry, R. A. affy - analysis of Affymetrix GeneChip data at the probe level. *Bioinformatics* **20**, 307-315, doi:10.1093/bioinformatics/btg405 (2004).
